# Hydrophobia Successfully Treated with Calomel

**Published:** 1860-03

**Authors:** 


*Hydrophobia Successfully Treated with Calomel.*

In the last number of the *American Journal of the Medical Sciences*, is the report of a case of hydrophobia, by Dr. J. E. H. Liggett, of Middleburgh, M. D., which recovered under the use of calomel, in the dose of one drachm every four hours, the intervals being diminished to six and eight hours, as the symptoms improved. Purgatives were also employed. There was only moderate salivation. The patient was a colored girl, 23 years old. Sixteen or eighteen days before she was taken sick, she had been bitten by a young dog, which had been unusually dull and morose for a day or two, who died afterwards with all the symptoms of rabies in its most virulant form. The symptoms began with pain in the great toe (the part bitten), extending up the limb toward the body. At the same time, from being a very lively girl, she became dull, moody, taciturn and irritable. The mind was clear, and she had frequent and violent spasms (of what muscle is not stated), which could at any time be excited by touching her, by a current of air, or by the sight of water or other fluids. There was intense thirst, but horror and immediate spasm from the sight of water; and expectoration of small quantities of viscid mucus. The pulse was of moderate frequency throughout. The medicine was followed by immediate relief, and the girl recovered in a week.

Dr. Ligget remarks that the diagnosis in the above case has been doubted, for two reasons: first, that the patient was a female, and secondly, that she recovered, the disease being supposed to be hysteria. Of course the fact that the patient recovered must throw some doubt on the nature of the disease, since there is no authentic record, so far as we know, of recovery from undoubted hydrophobia. We think, however, that the facts that the dog was mad, and that most of the symptoms of hydrophobia were present in the patient, are very strong reasons for believing that the case was one of genuine hydrophobia. At any rate it would be easy to try the remedy in another case.—*Boston Med. and Surg. Jour.*

